# Multi-objective optimization of mechanical properties in PLA/SCG/silane composites using synthetic data and XGBoost

**DOI:** 10.1039/d5ra06825h

**Published:** 2025-10-30

**Authors:** Atthaphon Ariyarit, Attasit Wiangkham, Phatthawit Siripaiboonsub, Jittiwat Nithikarnjanatharn, Wannisa Nutkhum, Prasert Aengchuan

**Affiliations:** a School of Mechanical Engineering, Institute of Engineering, Suranaree University of Technology Muang Nakhon Ratchasima 30000 Thailand; b Department of Industrial and Logistics Engineering, Faculty of Engineering, Srinakharinwirot University Ongkharak Nakhon Nayok 26120 Thailand; c Department of Industrial Engineering, Faculty of Engineering and Technology, Rajamangala University of Technology Isan Muang Nakhon Ratchasima 30000 Thailand; d School of Manufacturing Engineering, Institute of Engineering, Suranaree University of Technology Muang Nakhon Ratchasima 30000 Thailand prasert.a@sut.ac.th

## Abstract

Polylactic acid (PLA) composites reinforced with spent coffee grounds (SCG) and modified with a silane coupling agent (VTMS) offer a sustainable alternative for applications requiring biodegradability and enhanced mechanical performance. This study employed a data-driven approach to optimize tensile strength and Shore D hardness by varying the contents of PLA, SCG, and silane. Seventy-five composite samples were fabricated and tested, exhibiting tensile strengths of 26.5–57.9 MPa and hardness values of 77.5–80.8 Shore D. A multi-output XGBoost regression model, trained on 60% of the data and validated on the remaining 40%, achieved strong predictive accuracy (*R*^2^ = 0.884, MSE = 12.64 for tensile strength; *R*^2^ = 0.908, MSE = 0.071 for hardness) after augmentation with 159 synthetic samples generated *via* jittering, Gaussian noise, and kernel density estimation. Multi-objective optimization using NSGA-II simultaneously maximized both properties, revealing Pareto-optimal compositions dominated by higher PLA and moderate SCG and silane contents. The best formulation (1490 g PLA, 121 g SCG, 20 g silane) achieved 53.33 MPa tensile strength and 80.06 Shore D hardness. The combined XGBoost-NSGA-II framework demonstrates an efficient, data-driven strategy for optimizing bio-composite performance while minimizing experimental effort.

## Abstract 2

Polylactic acid (PLA) composites reinforced with spent coffee grounds (SCG) and modified with a silane coupling agent (VTMS) offer a sustainable alternative for applications requiring biodegradability and enhanced mechanical performance.

## Graphical abstract

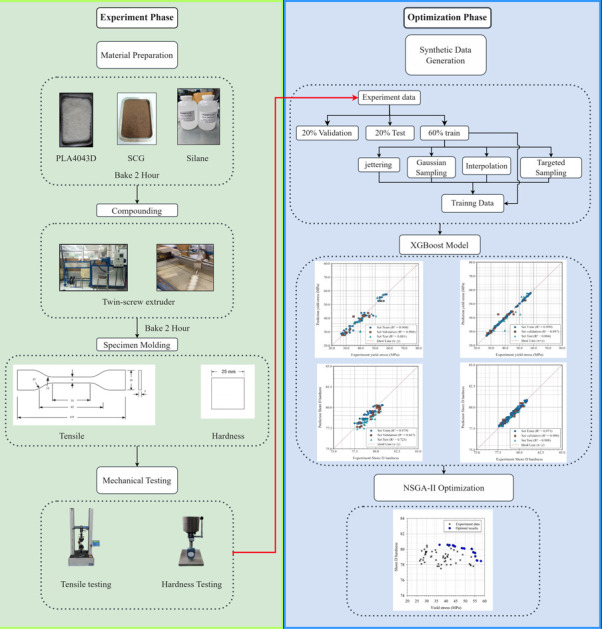

## 1 Introduction

Since the 1950s, the global accumulation of plastic waste has exceeded 8.3 billion metric tons. The COVID-19 pandemic in 2019 further intensified this issue by accelerating the consumption of disposable plastic-based protective equipment. Improper waste management contributes significantly to environmental pollution and poses serious threats to ecosystems and wildlife.^1–4^ In Thailand, a majority of polymers are still derived from petrochemicals and are not biodegradable, leading to long-term environmental impacts due to their resistance to natural degradation. Conventional waste disposal methods such as incineration, landfilling and mechanical recycling are energy intensive and can contribute to secondary pollution.^5–7^ In response, biodegradable polymers such as polylactic acid (PLA) have received increasing attention due to their compostability, environmental friendliness and compatibility with sustainable development goals.^8–10^ PLA can be synthesized from renewable feedstocks such as sugarcane and corn starch and is widely used in food packaging, biomedical devices and 3D printing applications.^11^

Despite its promising environmental profile, neat PLA suffers from inherent brittleness and limited impact resistance, which restrict its applications in load-bearing or high-stress environments. One common strategy to overcome these limitations involves reinforcing PLA with natural fillers. Among them, spent coffee grounds (SCG), a readily available, low-cost agricultural waste, have attracted attention for their ability to enhance the stiffness, thermal stability and biodegradability of PLA-based composites.^12–14^ Moreover, the use of silane coupling agents such as vinyltrimethoxysilane (VTMS) can improve the interfacial adhesion between the hydrophilic filler and hydrophobic PLA matrix, thus promoting efficient load transfer and enhancing mechanical properties.^15,16^ Several experimental studies and statistical design methods, including response surface methodology (RSM), have been employed to investigate the effects of SCG content and silane treatment on the mechanical performance of PLA composites.^17,18^ These properties are highly sensitive to formulation parameters such as filler content, particle dispersion and surface chemistry, necessitating precise control over composition and processing.^19,20^

Traditional trial-and-error experimentation is often time-consuming and inefficient. Recently, artificial intelligence (AI) and machine learning (ML) have been increasingly applied in materials research to model complex relationships between composition and properties.^21–25^ Recently, several studies have demonstrated the capability of ML models to accurately predict the mechanical behavior of polymer composites and optimize material performance. For example, Ulkir *et al.* employed artificial neural networks and fuzzy logic to predict the mechanical properties of 3D-printed PLA/wood composites,^26^ Omigbodun *et al.* utilized XGBoost and AdaBoost algorithms to model and enhance the mechanical performance of PLA/cHAP scaffolds for biomedical use,^27^ and Crupano *et al.* investigated 3D-printed PLA/PHB composites to support data-driven analysis of compressive and fatigue behavior.^28^ similarly, Fasikaw *et al.* demonstrated that AI models can successfully predict polymer composite behavior, while Lee *et al.* compared regression algorithms for metal forming, highlighting the superiority of tree-based methods.^29,30^ ML techniques such as linear regression, support vector machines, neural networks and tree-based algorithms have been applied to predict tensile strength, Shore D hardness and other critical properties. Among these, Extreme Gradient Boosting (XGBoost) has emerged as a robust and interpretable tool capable of handling nonlinear, high-dimensional data while providing feature importance metrics that offer practical insights into the influence of compositional factors such as PLA, SCG and silane.^31,32^

In many materials design problems, especially for multifunctional composites, multiple objectives such as tensile strength and surface Shore D hardness must be simultaneously optimized, which introduces trade-offs. Single-objective optimization approaches are insufficient in such scenarios. Consequently, multi-objective optimization algorithms like the Non-dominated Sorting Genetic Algorithm II (NSGA-II) have been widely adopted for their ability to efficiently explore large, multi-dimensional design spaces and generate a diverse set of Pareto-optimal solutions.^33–36^ NSGA-II was selected in this study over alternative methods such as multi-objective particle swarm optimization (MOPSO) and MOEA/D due to its strong balance between convergence speed and population diversity, as well as its proven effectiveness in discrete and high-dimensional composite optimization problems.^25^

In this study, we propose an integrated, data-driven framework that combines experimental testing, synthetic data generation, and multi-objective optimization. A multi-output XGBoost regression model is trained on both physical and synthetically augmented data to predict the tensile strength and Shore D hardness of PLA/SCG/Silane composites. Synthetic data are generated using techniques such as jittering, Gaussian noise injection, interpolation, and kernel density estimation (KDE), which enhance the diversity and coverage of the design space without compromising physical plausibility.

Unlike previous studies that primarily relied on linear regression or neural network models, this work leverages the XGBoost algorithm for multi-output prediction, which offers superior interpretability, fast convergence, and robustness to small and imbalanced datasets conditions often encountered in experimental materials research. The integration of synthetic data generation with XGBoost enables effective learning from limited samples, while the feature-importance metrics provide quantitative insights into the influence of each compositional factor. The trained surrogate model is further embedded into the NSGA-II framework to efficiently identify Pareto-optimal composite formulations. This combined approach establishes a scalable, accurate, and cost-effective pathway for the rational design and optimization of high-performance, sustainable bio-composite materials.

## 2 Research & methods

**Fig. 1:**
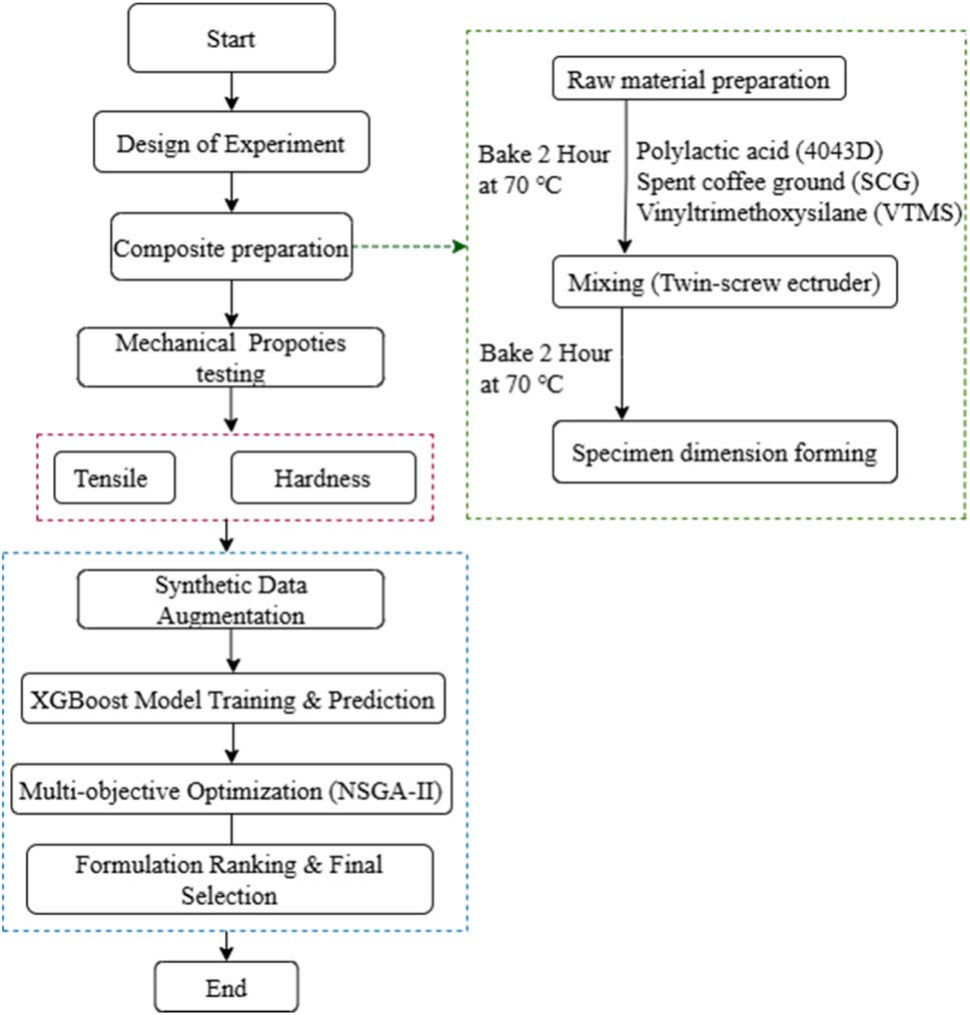
Workflow diagram.

This study aims to systematically investigate the effects of filler content and surface treatment on the mechanical properties of PLA based bio-composites. A structured methodology was employed, combining experimental formulation with machine learning-based optimization, as illustrated in Fig. 1. The process began with the preparation of raw materials, including polylactic acid (PLA), spent coffee grounds (SCG) and vinyltrimethoxysilane (VTMS) as a silane coupling agent. These materials were mixed using a twin-screw extruder and subsequently formed into test specimens with standardized dimensions. Mechanical performance was assessed through tensile strength and Shore D hardness tests. To enhance predictive accuracy and explore a broader formulation space, synthetic data augmentation was applied. A multi-output XGBoost regression model was trained using both original and synthetic data to predict the mechanical responses. Multi-objective optimization was then conducted using the Non-dominated Sorting Genetic Algorithm II (NSGA-II) to identify optimal formulations. The entire workflow from experimental design and material preparation to predictive modeling and optimization is summarized in Fig. 1.

### 2.1 Design of experiment

To systematically investigate the effects of material composition on the mechanical properties of PLA/SCG/Silane bio-composites, this study employed a central composite design (CCD), a widely accepted experimental design under the response surface methodology (RSM) framework. CCD enables the efficient construction of predictive models while reducing the number of required experiments and it allows for the evaluation of linear, interaction and quadratic effects among the selected factors.^37–39^ This design strategy was particularly suitable for the present study, which operated under budgetary constraints that limited the total number of experimental trials that could be practically conducted.

Based on the CCD approach, 15 distinct formulations were generated and each was replicated 5 times, resulting in a total of 75 composite samples for mechanical testing. Three independent variables PLA, SCG and silane, as shown in Table 1.

**Table 1:** Composite material formula from CCD

PLA (g)	SCG (g)	Silane (g)
1409	359	60
1275	225	75
1141	359	15
1409	359	15
1141	91	60
1275	0	38
1275	225	38
1050	225	38
1500	225	38
1275	225	0
1275	450	38
1409	91	15
1409	91	60
1141	91	15
1141	359	60

### 2.2 Composite preparation

**Fig. 2:**
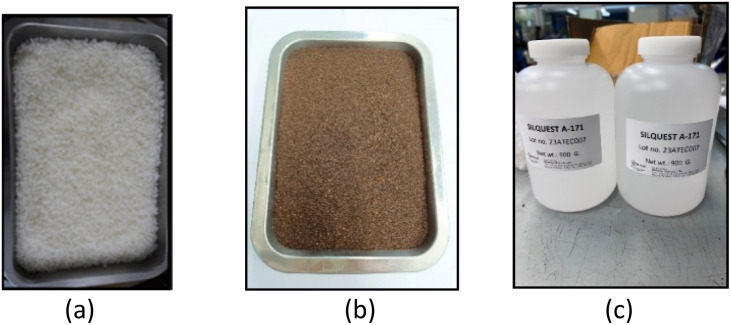
Raw materials used for composite preparation: (a) polylactic acid (PLA); (b) spent coffee grounds (SCG); and (c) vinyltrimethoxysilane (VTMS).

In the specimen preparation process, PLA based bio-composites were formulated using polylactic acid (PLA, grade 4043D) as the polymer matrix, spent coffee grounds (SCG, 80 mesh) as a bio-based filler and vinyltrimethoxysilane (VTMS) as a coupling agent to enhance interfacial adhesion between the matrix and filler. Prior to processing, PLA and SCG were dried separately in a hot air oven at 70 °C for 2 hours to eliminate residual moisture, as shown in Fig. 2(a–c). The silane coupling agent was not subjected to the drying process to prevent premature hydrolysis or volatilization. The dried components were then manually dry blended, using 100 grams of PLA per tray and adding SCG and silane according to the target formulation concentrations.

**Fig. 3:**
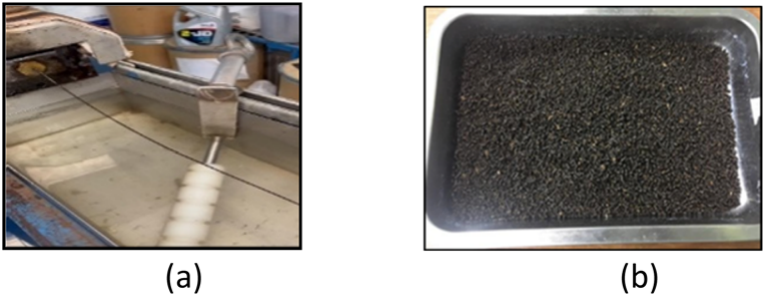
Extruded bio-composite materials at different processing stages: (a) continuous strands from twin-screw extrusion; (b) pelletized granules after mechanical grinding.

The blended materials were subsequently compounded using a twin-screw extruder operated at 180 °C under controlled thermal and shear conditions to ensure uniform dispersion and promote chemical interaction between the PLA matrix and the surface treated filler. The extruded material initially emerged in the form of continuous strands, as shown in Fig. 3(a), which were then cooled and mechanically pelletized into granules, as illustrated in Fig. 3(b). These pellets were subjected to an additional drying cycle under the same conditions prior to molding.

Importantly, the extrusion process was performed sequentially, beginning with the formulation containing the lowest SCG content and gradually progressing to those with higher filler loadings. This order of processing was implemented to minimize filler carry over and prevent cross contamination between different formulations.

### 2.3 Mechanical properties testing

**Fig. 4:**
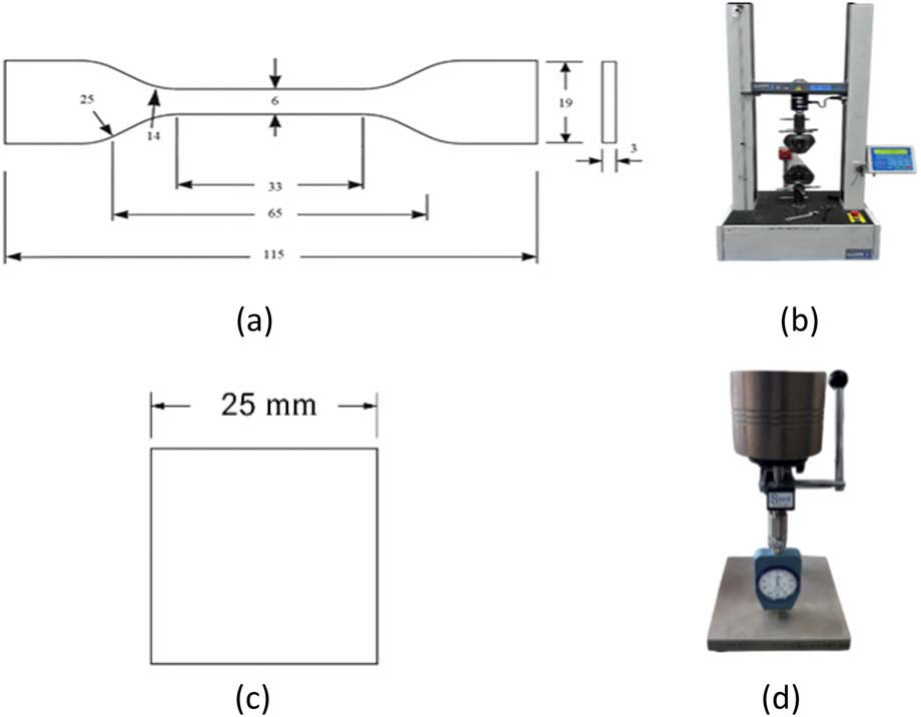
(a) Dimensions of the test specimen for tensile testing; (b) tensile testing machine; (c) dimensions of the Shore D hardness test specimen; and (d) Shore D durometer.

The mechanical properties testing in this study involved two types of equipment: a tensile testing machine and a Shore D hardness tester. Tensile tests were conducted in accordance with ASTM D638 Type IV^40^ using a LLOYD LR10K Plus universal testing machine at a constant crosshead speed of 50 mm min^−1^, as shown in Fig. 4(b). Hardness measurements were carried out based on ASTM D2240 (ref. 41) using a Shore D durometer with a 10 lbf (approximately 4.5 kgf) applied load, as illustrated in Fig. 4(d). Each test was performed on five replicate specimens per formulation and the average value was reported for both tensile strength and Shore D hardness to ensure reliability and reproducibility.

Tensile test specimens were fabricated using a vertical injection molding machine, which provides high precision and consistency in specimen formation. The geometry of the tensile specimens followed the ASTM D638 Type IV standard.^40,42,43^ as shown in Fig. 4(a) presents the specimen dimensions in millimeters (mm), which is widely used for testing thermoplastics and composite materials with limited thickness.

For Shore D hardness testing, the specimens were prepared and measured in accordance with the ASTM D2240 standard,^41–44^ as illustrated in Fig. 4(c). The specimens used for this test were cube in shape, with equal side lengths to ensure consistent contact with the Shore D hardness indenter. This method allows for consistent and reliable assessment of surface hardness in rigid plastic materials.

After molding, all specimens were conditioned at room temperature for 48 hours to relieve residual stresses and stabilize dimensional properties prior to mechanical testing.

### 2.4 Multi objective optimization

The development of bio-composite materials with enhanced mechanical properties often requires simultaneous optimization of multiple performance criteria, such as tensile strength and surface hardness. In such cases, improving one property may compromise another, leading to a need for multi-objective optimization strategies that can effectively explore tradeoffs and identify balanced solutions. Conventional optimization techniques, such as trial and error experimentation or single objective tuning, are typically insufficient for navigating the complex and nonlinear relationships inherent in multicomponent composite systems.

To address these challenges, this study proposes an integrated framework that leverages artificial intelligence (AI) for property prediction and evolutionary algorithms for multi-objective optimization. Specifically, Extreme Gradient Boosting (XGBoost) was adopted as the surrogate model due to its high accuracy, robustness to overfitting and ability to handle complex nonlinear datasets.^45,46^

**Fig. 5:**
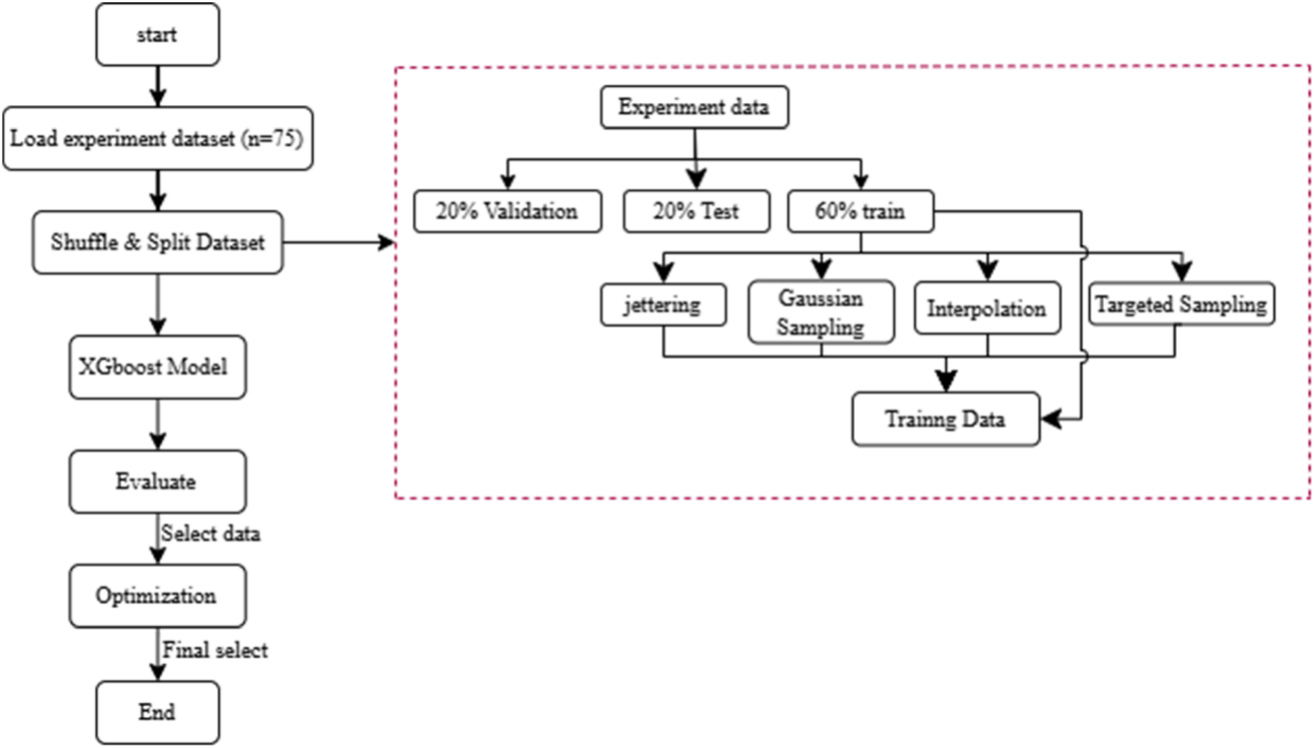
Workflow of the AI-assisted multi-objective optimization framework integrating XGBoost prediction and synthetic data augmentation.

The experimental data used to train the model were generated using central composite design (CCD), a widely used statistical approach under the response surface methodology (RSM) framework. While CCD provides an efficient means of exploring factor interactions with a limited number of experimental runs, the resulting dataset often lacks sufficient coverage of the full compositional space. To mitigate this limitation and enhance the generalization capacity of the surrogate model, synthetic data were generated using statistical augmentation techniques including jittering, Gaussian noise injection, interpolation and targeted sampling of low-density regions.^47,48^ These synthetic samples preserved the statistical structure and physical plausibility of the original data while significantly increasing the diversity of training inputs. The overall workflow of the proposed data-driven optimization process, integrating experimental data, synthetic augmentation, XGBoost modeling, and NSGA-II optimization, is illustrated in Fig. 5.

The augmented dataset was then used to train a multi-output XGBoost model capable of predicting both tensile strength and Shore D hardness based on the composite formulation inputs: PLA, SCG and silane content. Once trained, the XGBoost model was embedded within the Non-dominated Sorting Genetic Algorithm II (NSGA-II) framework to perform multi-objective optimization. The goal was to simultaneously maximize both tensile strength and Shore D hardness, with the surrogate model guiding the search over a wide design space without requiring additional physical experiments. This integrated approach offers a scalable, data driven pathway for discovering optimal bio-composite formulations and advancing sustainable material development.

Additional mathematical formulations and detailed algorithmic procedures for data augmentation, XGBoost training, and NSGA-II optimization are provided in the SI.

## 3 Result and discussion

This chapter presents and discusses the results of the study, beginning with the analysis of experimental data obtained from the fabrication and mechanical testing of PLA/SCG/Silane bio-composites. The experimental dataset, which includes variations in PLA, spent coffee grounds (SCG) and silane content, serves as the foundation for modeling the mechanical performance of the composites.

To enhance the predictive capability of the machine learning model, synthetic data were subsequently generated and combined with the original experimental dataset. This augmented dataset was used to train and compare the performance of XGBoost models, enabling a direct evaluation of whether the inclusion of synthetic data improves model accuracy and generalization. The performance of both models trained on original data and on the augmented dataset was assessed based on standard metrics, including *R*^2^ and MSE, for both tensile strength and Shore D hardness.

Following model evaluation, the optimized model was integrated into the Non-dominated Sorting Genetic Algorithm II (NSGA-II) to perform multi-objective optimization. The optimization aimed to simultaneously maximize tensile strength and Shore D hardness, generating a well-distributed set of Pareto-optimal solutions. To support the decision-making process, a composite performance score calculated from normalized tensile strength and Shore D hardness was used to rank the solutions and identify the most well-balanced formulations.

Finally, the top five optimized formulations with the highest composite scores are presented and discussed, providing practical guidelines for selecting compositions that achieve an optimal balance between mechanical performance and material efficiency.

### 3.1 Mechanical properties result of PLA composite

**Fig. 6:**
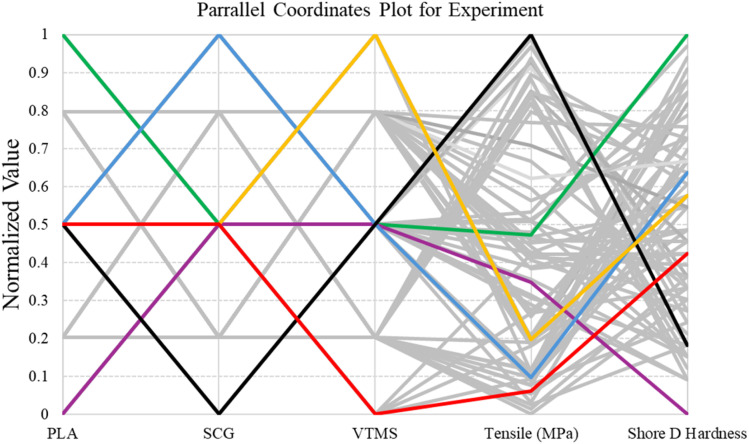
Parallel coordinates plot for experimental data.

To investigate the relationships among the input parameters and mechanical responses, a parallel coordinates plot (PCP) was constructed, as shown in Fig. 6. This plot provides a clear visual representation of how different levels of PLA, SCG and silane content influence the tensile strength and Shore D hardness of the composite specimens. Each line represents a single experimental run, connecting normalized values of each variable across the vertical axes. Highlighted lines indicate selected samples of interest.

The plot reveals several notable trends across the dataset. Specimens with high PLA content generally exhibited higher tensile strength, suggesting that PLA plays a dominant role in structural reinforcement. This is clearly illustrated by the green and purple lines, where higher PLA levels correspond to increased tensile strength and Shore D hardness. Conversely, when PLA content is low as seen in the same green and purple lines the values for both mechanical properties tend to decrease. SCG content, on the other hand, showed an inverse trend. As indicated by the blue and black lines, increasing SCG content is associated with a reduction in tensile strength, likely due to the lower stiffness and poor bonding ability of spent coffee grounds. Interestingly, these lines also reveal that higher SCG levels can slightly enhance Shore D hardness, while lower SCG levels tend to support higher tensile strength but lower Shore D hardness values. The effect of silane (VTMS) is more nuanced. The yellow and red lines show that increasing silane content tends to improve both tensile strength and Shore D hardness, particularly at moderate to high dosages. This trend suggests that silane enhances interfacial bonding between PLA and SCG, contributing to better stress transfer and mechanical performance.

Overall, the PCP plot provides insight into how compositional factors influence the performance of the composites, helping to identify input combinations that yield favorable mechanical outcomes and guiding future model development and optimization efforts.

### 3.2 Multi-objective optimization result

Before conducting the multi-objective optimization process, the predictive performance of the XGBoost regression model was thoroughly evaluated using both the original experimental dataset and the augmented dataset, which incorporated 159 synthetic data points. The inclusion of synthetic data aimed to improve model generalization by expanding feature space and mitigating overfitting, particularly under limited experimental data.

**Fig. 7:**
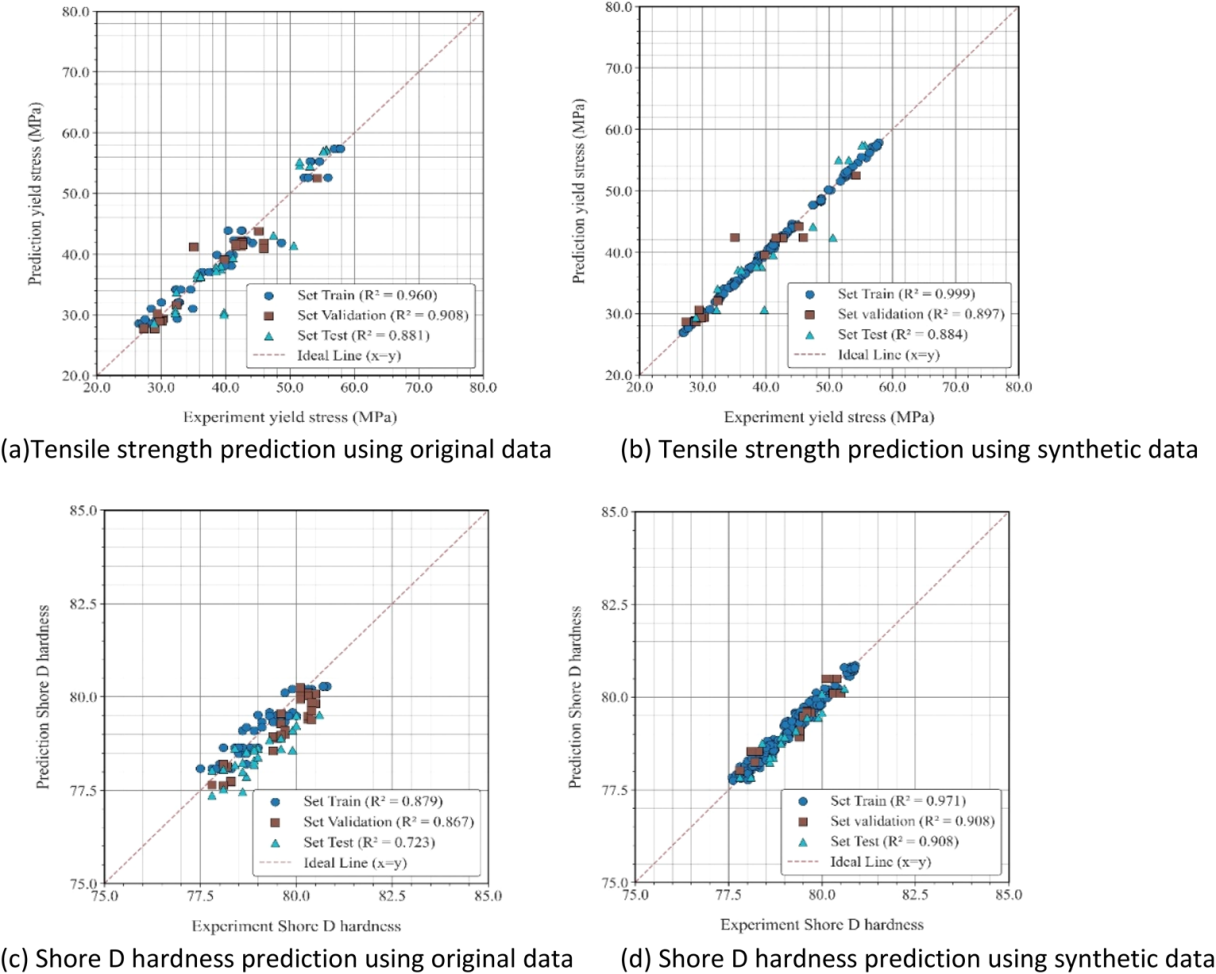
Comparison of predicted and experimental values for tensile strength and Shore D hardness using original and synthetic datasets. (a)Tensile strength prediction using original data (b) tensile strength prediction using synthetic data (c) Shore D hardness prediction using original data (d) Shore D hardness prediction using synthetic data.

The model's performance was assessed in terms of the coefficient of determination *R*^2^ and mean squared error (MSE) for both tensile strength and Shore D hardness. As shown in Figures 7(a)–(d), the predicted values are closely aligned with the experimental results, with most data points distributed along the 45° reference line. For tensile strength, the model trained with synthetic data achieved a slightly higher test *R*^2^ of 0.884 and a lower MSE of 12.641, compared to 0.881 and 13.608 for the model trained on original data. A more substantial improvement was observed in the Shore D hardness prediction, where *R*^2^ increased from 0.723 to 0.908, and MSE decreased markedly from 0.431 to 0.071 after data augmentation. These results confirm that the addition of synthetic data enhanced the model's predictive accuracy and consistency, particularly for hardness prediction, which initially exhibited greater variability. Such improvements echo findings in other materials prediction studies using XGBoost and data augmentation.^49^

Table 2 presents the results of the 5-fold cross-validation conducted to evaluate the stability and generalization of the XGBoost model. Each fold used 80% of the data for training and 20% for validation. The synthetic-augmented model achieved a slightly higher average *R*^2^ value (0.859) compared to the model trained on original data (0.837), indicating improved predictive consistency across folds. Although the average MSE increased marginally (4.171 *vs.* 3.576), the overall variation among folds was reduced, suggesting that the inclusion of synthetic data enhanced model robustness and reduced overfitting. This behavior aligns with literature reporting that boosting algorithms are effective for small or imbalanced datasets when augmented training data are available.^50,51^

**Table 2:** Summary of k-fold cross-validation performance metrics (*R*^2^ and MSE) for models trained on original and synthetic data

K fold (run fold)	Average *R*^2^ macro (original data)	Average MSE macro (original data)	Average *R*^2^ macro (synthetic data)	Average MSE macro (synthetic data)
Run 1 (fold = 1)	0.893	3.400	0.881	3.469
Run 2 (fold = 2)	0.745	2.751	0.887	3.347
Run 3 (fold = 3)	0.816	6.416	0.841	4.692
Run 4 (fold = 4)	0.839	2.224	0.793	4.937
Run 5 (fold = 5)	0.892	3.090	0.891	4.409
Average	0.837	3.576	0.859	4.171

**Fig. 8:**
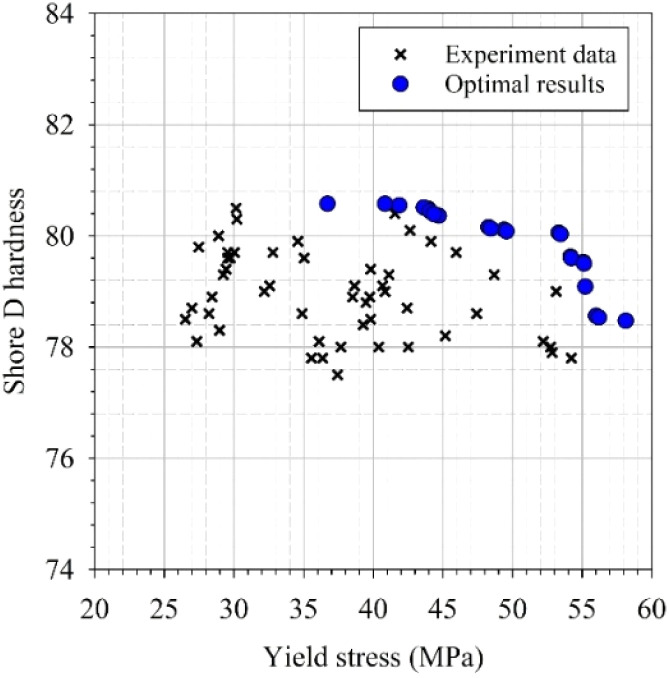
Comparison of experimental data and NSGA-II Pareto-optimal solutions for tensile strength and Shore D hardness.

Based on these findings, the synthetic-augmented XGBoost model was selected as the surrogate model for subsequent NSGA-II multi-objective optimization, providing a balanced trade-off between prediction accuracy and stability across different data partitions. To further optimize the mechanical performance of PLA/SCG/Silane bio-composites, the NSGA-II algorithm was employed to simultaneously maximize tensile strength and Shore D hardness. The resulting Pareto-optimal front, shown in Fig. 8, reveals a smooth trade-off between the two objectives, with optimal formulations concentrated in regions of higher tensile strength while maintaining or slightly improving hardness. The use of NSGA-II for exploring trade-offs in composite design is well established in engineering and materials optimization literature.^52,53^ These results indicate that increasing PLA content while maintaining moderate SCG and silane levels yields superior overall mechanical performance, offering practical guidance for designing composite formulations that meet specific application requirements.

To facilitate decision-making among the Pareto-optimal solutions, a set of composite score formulas was introduced to evaluate the overall performance of each candidate. These composite scores combine tensile strength and Shore D hardness into a single performance index using different weighting strategies and mathematical models. Five representative formulations obtained from the NSGA-II optimization are summarized in Table 3, showing their corresponding tensile strength, Shore D hardness, and composite scores. The composite score serves as an integrated indicator that balances both mechanical properties, allowing the identification of the most optimal formulation.

**Table 3:** Optimized composite formulations and composite scores obtained from NSGA-II

PLA (g)	SCG (g)	Silane (g)	Tensile (MPa)	Shore D hardness	Composite Score
1490	121	20	53.33	80.06	1.5282
1426	121	20	53.46	80.03	1.5214
1496	162	20	49.40	80.11	1.3703
1426	162	20	49.57	80.08	1.3651
1496	60	20	54.17	79.62	1.3613

The composite score was introduced as a normalized performance index to evaluate the overall mechanical quality of each formulation by simultaneously considering both tensile strength and Shore D hardness. It was calculated as the sum of the normalized values of these two properties, allowing a balanced comparison between formulations with different trade-offs. A higher composite score indicates better combined mechanical performance.

Table 3 summarizes the representative composite formulations obtained from the NSGA-II optimization process, along with their corresponding tensile strength, Shore D hardness, and composite scores. In these optimized formulations, the silane content (VTMS) was fixed at 20 g, while the PLA and SCG contents were varied to study their combined influence on mechanical behavior. A detailed mathematical definition and normalization procedure used for computing the composite score are provided in the SI for completeness and reproducibility.

The results indicate that formulations with lower SCG content tend to achieve higher composite scores and better mechanical properties. The highest composite score of 1.5282 was obtained for the formulation containing 1490 g PLA, 121 g SCG, and 20 g Silane, which exhibited a tensile strength of 53.33 MPa and a Shore D hardness of 80.06. A similar formulation with 1426 g PLA and the same SCG and silane levels followed closely with a score of 1.5214.

Conversely, increasing the SCG content to 162 g caused a decline in tensile strength to 49.40–49.57 MPa despite a slight gain in hardness, resulting in lower composite scores (1.3703–1.3651). This suggests that excessive SCG weakens the structural integrity of the composite due to its lower intrinsic strength compared with the PLA matrix.

Interestingly, the formulation achieving the highest tensile strength of 54.17 MPa and a hardness of 79.62 contained only 60 g of SCG, yielding a high composite score of 1.3613. These observations confirm that moderate filler loading enhances the overall mechanical performance of PLA-based bio-composites.

### 3.3 Data preparation and synthetic data augmentation

To construct an accurate and generalizable predictive model for composite property estimation, this study implemented a multi-output Extreme Gradient Boosting (XGBoost) regressor. The model was trained to predict two key mechanical responses tensile strength (MPa) and Shore D hardness based on three input features: PLA content (g), spent coffee grounds (SCG, g) and silane concentration (g).

The original dataset comprised 75 experimentally derived samples generated using a Central Composite Design (CCD) approach. Although CCD efficiently captures interactions and quadratic effects with a reduced number of experiments, its limited coverage of the input space poses challenges for training machine learning models with high generalization capability. Therefore, the dataset was randomly shuffled and partitioned into three distinct subsets: 60% training set: used for model fitting and data augmentation, 20% validation set: used for hyperparameter tuning and performance monitoring, 20% test set: reserved for final model evaluation. synthetic data augmentation was applied exclusively to the training subset to prevent information leakage. Four techniques were employed to generate synthetic samples:^54–57^

(1) Jittering eqn (1): small Gaussian noise is added to both the input and output variables to create local perturbations around the original training points:

1 *X̃* = *X* + *ε*_*X*_, *Ỹ* = *Y* + *ε*_*Y*_

where *X̃* represents the synthesized input feature vector after noise injection, *X̃* is the original input vector (*e.g.*, PLA, SCG and Silane content), *ε*_*X*_ is a small random noise drawn from a normal distribution and *Ỹ*, *Y* and *ε*_*Y*_ ∼ *N*(0, *σ*^2^) are the corresponding output vector, actual value and noise respectively.

(2) Gaussian Sampling eqn (2): new samples are drawn from a multivariate Gaussian distribution based on the mean and covariance of the original dataset:

2 *X̃* ∼ *N*(*μ*_*X*_, Σ_*X*_), *Ỹ* = *Y*_nearest_ + *ε*_*Y*_

where *X̃* represents the input vector synthesized by drawing samples from a multivariate normal distribution with mean*μ*_*X*_ and covariance matrix Σ_*X*_ calculated from the original dataset, *Y*_nearest_ is the output value of the nearest neighbor in the training set and *ε*_*Y*_ ∼ *N*(0, *σ*^2^) is small noise added to preserve variability.

(3) Interpolation eqn (3): new data points are synthesized by convex combinations of randomly selected input pairs:

3 *X̃* = *αX*_*i*_ + (1 − *α*)*X*_*j*_, Ỹ = *αY*_*i*_ + (1 − *α*)*Y*_*j*_

where *X̃* is the new input vector created by interpolating between two randomly selected input vectors *X*_*i*_ and *X*_*j*_ from the training data,α ∈ [0, 1] is a randomly chosen interpolation coefficient and *Ỹ* is the interpolated output vector from corresponding values *Y*_*i*_ and *Y*_*j*_.

(4) Targeted Sampling eqn (4): low-density regions in the input space, identified by kernel density estimation (KDE), are perturbed to create new samples:

4 *X̃* = *X*_*k*_ + *δ*_*X*_, *Ỹ* = *Y*_*k*_ + *δ*_*Y*_

where *X̃* is an input vector located in a low density region of the training space as identified by kernel density estimation, *δ*_*X*_ ∼ *N*(0, *τ*^2^) is perturbation applied to the input and *Ỹ* is the corresponding synthesized output value obtained by adding small noise *δ*_*Y*_ to the actual target *Y*_*k*_.

The synthetic data generated from the training set were combined with the original training data to form an augmented dataset, while the test set remained untouched for independent validation.

### 3.4 XGBoost model construction

**Fig. 9:**
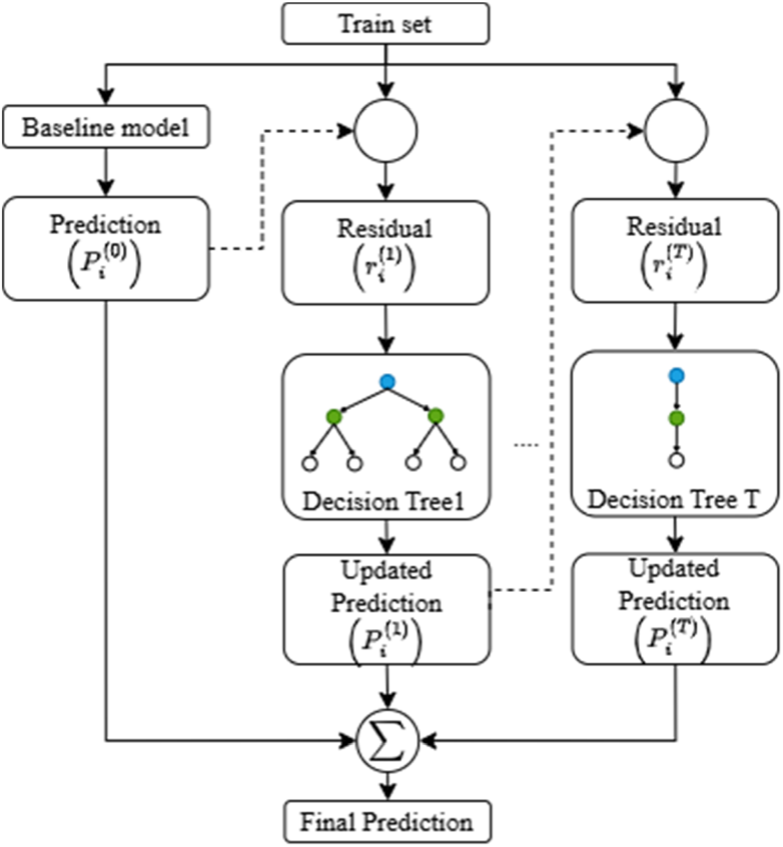
XGBoost workflow.

To develop a predictive model capable of accurately estimating both tensile strength and Shore D hardness of PLA/SCG/Silane composites, the Extreme Gradient Boosting (XGBoost) algorithm was adopted as the regression model. This ensemble learning method builds a strong learner by iteratively combining multiple weak learners typically decision trees with each tree trained to minimize the residual errors of the previous iterations,^58,59^ as illustrated in Fig. 9.^46^

At the beginning of the learning phase, the model initializes with a baseline prediction, *P*^(0)^, which is typically the mean value of each target variable (tensile strength and Shore D hardness) in the training set, as shown in eqn (5):5
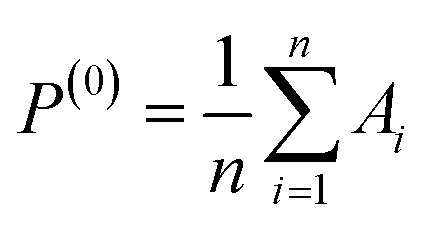
where *P*^(0)^ represents the initial prediction at iteration zero, *A*_*i*_ is the actual value of the target variable (tensile strength or Shore D hardness) for data point *i* in the training set and *n* denotes the total number of data points used in model training.

Following this initialization, residuals are computed at each *t* iteration to quantify the error between actual and predicted values. These residuals guide the learning of subsequent trees, as shown in eqn (6):

6 *r*_*i*_^(*t*)^ = *A*_*i*_ − *P*_*i*_^(*t*)^

where *r*_*i*_^(*t*)^ represents the residual (prediction error) of data point *i* at the iteration *t*, *A*_*i*_ is the actual value and *P*_*i*_^(*t*)^ denotes the prediction of the model for sample at the same *i* iteration

A regression tree *h*^(*t*)^(*x*_*i*_) is trained to minimize the squared error between the predicted residuals and the actual residuals from the previous iteration, as shown in eqn (7):7
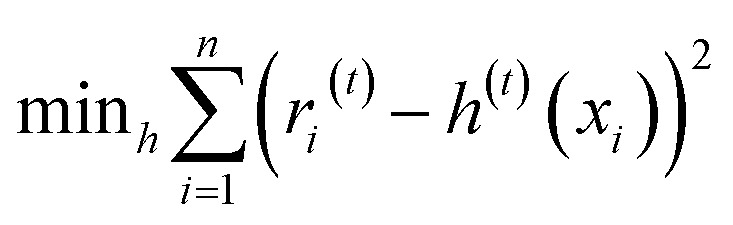
where *r*_*i*_^(*t*)^ is the residual at iteration *t*, *h*^(*t*)^(*x*_*i*_) is the output of the new regression tree and *n* the number of training samples.

Once the regression tree is fitted, the model undergoes optimization through the regularized objective function, which balances the prediction error and model complexity. This optimization process is guided by gradient boosting principles and is formulated in eqn (8):8
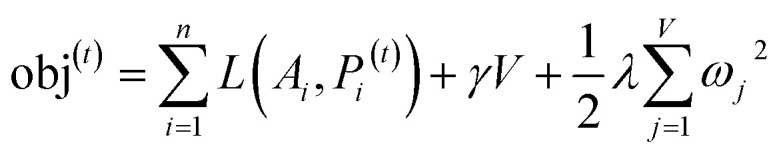
where obj^(*t*)^ is the objective function value at iteration *t* , *L*(*A*_*i*_, *P*_*i*_^(*t*)^) denotes the loss function, such as squared error, between the actual value *A*_*i*_ and the predicted value *P*_*i*_^(*t*)^, *V* represents the number of leaves in the regression tree, *ω*_*j*_ is the weight assigned to leaf *j*, *γ* is the regularization parameter that penalizes excessive tree complexity and *λ* controls the magnitude of leaf weights.

After optimizing the objective function, the model is updated by incorporating the output of the newly fitted regression tree. This update adjusts the previous prediction by adding a scaled contribution from the new tree, as shown in eqn (9):

9 *P*_*i*_^(*t*+1)^ = *P*_*i*_^(*t*)^ + *η* × *h*^(*t*)^(*x*_*i*_)

where *P*_*i*_^(*t*+1)^ is the updated prediction for data point *i*, *η* is the learning rate, which controls the contribution of each tree and *h*^(*t*)^(*x*_*i*_) is the predicted residual for data point *i* from the regression tree trained in iteration *t*.

This iterative learning process continues until the predefined number of boosting rounds (iterations) is reached or until convergence criteria are satisfied. The final prediction of the model after *T* iterations is obtained by aggregating the contributions from all individual trees, as shown in eqn (10):10
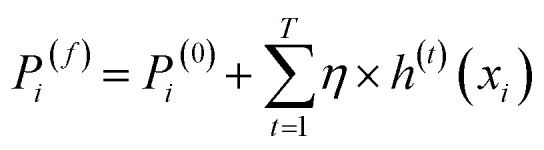
where *P*_*i*_^(*f*)^ represents the final predicted value for data point *i* and all other variables are defined as previously.

According to the XGBoost learning framework, the predictive performance of the model is highly dependent on several hyperparameters that govern how the model fits the data, manages complexity and avoids overfitting. In this study, key hyperparameters were optimized using Optuna with the Tree-structured Parzen Estimator (TPE) sampler. The search space included the number of estimators (ranging from 100 to 500), maximum tree depth (3 to 8), learning rate (0.001 to 0.1 on a logarithmic scale), subsample ratio (0.6 to 1.0), column sampling ratio per tree (0.6 to 1.0), gamma (0 to 0.4) for minimum loss reduction required to make a split and minimum child weight (1 to 4), which controls the minimum sum of instance weights needed in a child node. A total of 50 optimization trials were performed, with validation set mean squared error (MSE) used as the evaluation metric. The best-performing hyperparameter configuration was then used to train the final XGBoost model on the combined training and validation sets. This systematic optimization strategy effectively enhanced model generalization and predictive robustness across both target properties.

To assess the predictive performance of the XGBoost surrogate model constructed in this study, two widely accepted statistical metrics were employed: the coefficient of determination (*R*^2^) and the mean squared error MSE. These regression evaluation metrics were used to quantify the model's ability to accurately estimate the mechanical properties (tensile strength and Shore D hardness) based on input features (PLA, SCG and silane content).

The coefficient of determination, denoted as *R*^2^, measures the proportion of the variance in the dependent variable (*i.e.*, experimental data) that is predictable from the independent variables (*i.e.*, model predictions). The *R*^2^ value ranges from negative infinity to 1, where a value closer to 1 indicates a better fit.^60^ The mathematical formulation of *R*^2^ is shown in eqn (11)11
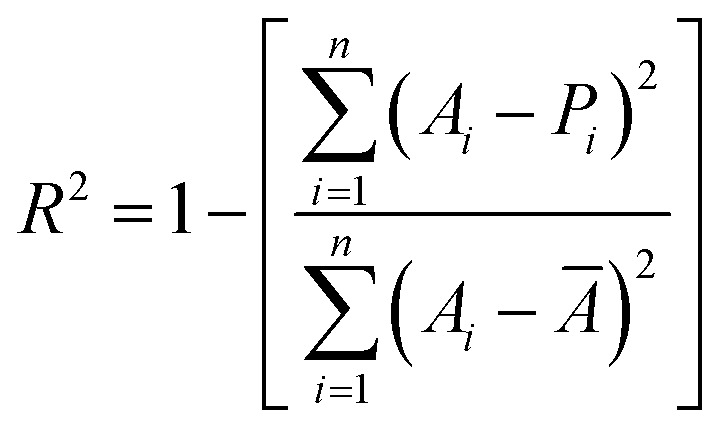
where *A*_*i*_ denotes the actual value of the target variable at the *i* sample, *P*_*i*_ is the predicted value and *Ā* is the mean of actual values. A higher *R*^2^ indicates that the model captures more variance and provides better predictions.

The second metric, mean squared error (MSE), evaluates the average of the squared differences between predicted and actual values, placing greater weight on larger errors.^61^ The formulation of MSE is given in eqn (12):12
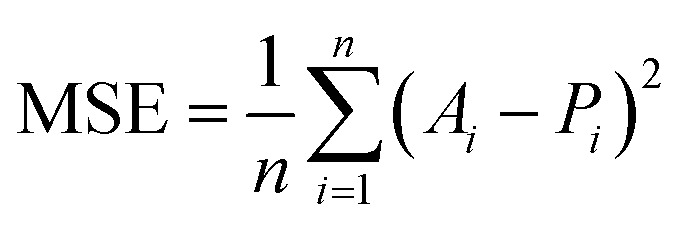
where *n* is the number of points. A lower MSE value indicates a smaller average error and therefore better model performance.

### 3.5 Multi-objective optimization

The complex interplay between mechanical properties such as tensile strength and Shore D hardness in PLA/SCG/Silane bio-composites requires a careful balance in material formulation. Improving one property may lead to trade-offs in the other, thus necessitating the use of multi-objective optimization techniques to simultaneously address multiple design targets.

To effectively address this challenge, the present study employed the Non-dominated Sorting Genetic Algorithm II (NSGA-II), a widely recognized multi-objective evolutionary algorithm. NSGA-II is particularly valued for its ability to maintain solution diversity (population heterogeneity) while converging toward the Pareto-optimal front. Its robust performance and computational efficiency have led to its successful application across a range of domains, including materials engineering. In this study, NSGA-II was implemented to simultaneously maximize the tensile strength and Shore D hardness of PLA-based bio-composites. The corresponding optimization problem was defined with two objectives and three decision variables (inputs), as outlined in eqn (13) and (14).13
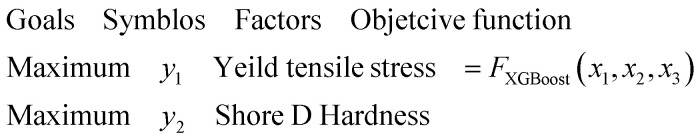
14
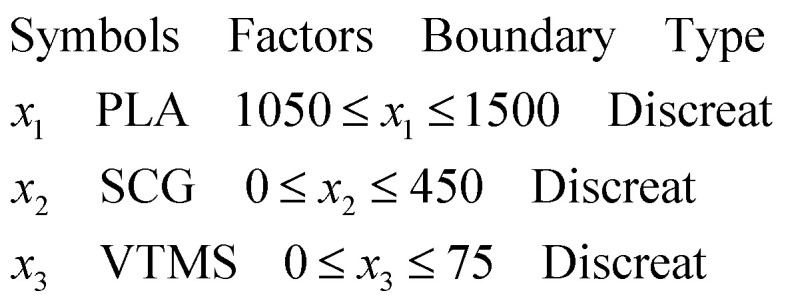
In the first step, the initialization of a population of candidate solutions (individuals) was performed. Each individual represents a possible combination of PLA, SCG and Silane was randomly initialized within the defined bounds, as shown in eqn (15).

15 *X⃑*_*i*_ = [*x*_1_, *x*_2_, *x*_3_]

where *X⃑*_*i*_ refers to the *i* in the population. It is a vector of decision variables used to predict the objective function values.

In step 2, evaluation, each individual in the population was evaluated using a pre-trained XGBoost model to predict the values of the two objective functions: tensile strength and Shore D hardness. Since the DEAP framework performs minimization by default, the predicted values were negated in the fitness function, as shown in eqn (16)

16 *f*_1_(*X*_*i*_) = −*y*_1_ , *f*_2_(*X*_*i*_) = −*y*_2_

In Step 3, non-dominated Sorting was applied to rank individuals based on Pareto dominance. Each solution in the population was compared pairwise to determine whether it is dominated by or dominates others, according to the following condition: a solution A dominates solution B if it is no worse in all objectives and strictly better in at least one. This classification resulted in a hierarchy of Pareto fronts, where the first front (*F*_1_) consists of non-dominated solutions and subsequent fronts contain solutions that are dominated by those in the preceding fronts. To ensure population diversity, a crowding distance metric was applied to each front. The crowding distance quantifies the density of solutions in the objective space by estimating the proximity of each individual to its neighbors. For each individual *i*, the crowding distance *d*_*i*_ was calculated using normalized distances across all objective functions, as defined in eqn (17):17
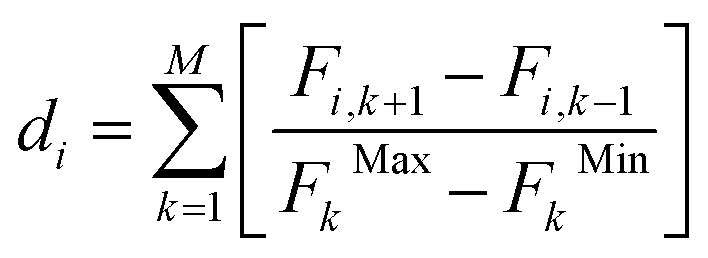
where *d*_*i*_ is the crowding distance of individual *i*, *F*_*i*,*k*+1_ and *F*_*i*,*k*−1_ represent the values of the *k* objective for the next and previous individuals in the sorted list, *F*_*k*_^Max^ and *F*_*k*_^Min^ are the maximum and minimum values of objective *k* within the same front. This measure ensured that individuals located in less crowded regions of the objective space were favored, thereby preserving diversity as the population evolved.

In Step 4, Genetic Operations, once the solutions were sorted based on Pareto dominance and evaluated for diversity using crowding distance, genetic operators such as selection, crossover and mutation were applied to generate new offspring. The selection process was performed using a binary tournament selection method, which selects the fittest individuals based on their non-dominated rank and crowding distance, ensuring a balance between convergence and diversity.

Crossover and mutation were then employed to introduce variation into the population. The crossover operation combines the genetic information of two parent individuals to produce new offspring, while mutation introduces small random perturbations to a single individual. These operations allow the algorithm to explore new regions of the solution space and potentially discover better-performing combinations than those in the previous generation. the two genetic operations can be expressed as follows in eqn (18) and (19)18
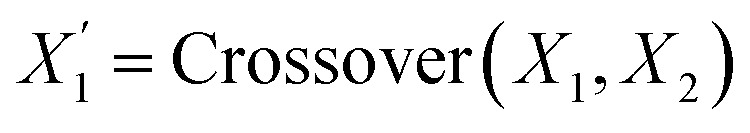
19
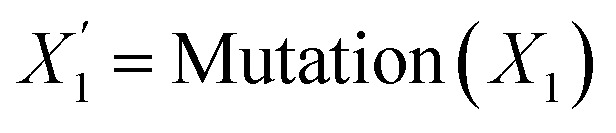
where *X*_1_ and *X*_2_ are parent individuals and 
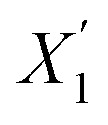
 is the resulting offspring. The crossover was implemented using uniform crossover with a probability of 0.5 and mutation was performed using uniform integer mutation with a per-gene probability of 0.2, within the predefined bounds of each variable.

In Step 5, After generating the offspring population through crossover and mutation, the algorithm proceeded to the survival selection phase. In this step, the parent and offspring populations were merged to form a combined population of double the original size. Non-dominated sorting was then reapplied to this combined population to reclassify all individuals into updated Pareto fronts. Selection was performed based on two primary criteria: (1) Pareto rank, where individuals in lower-ranked fronts are preferred and (2) crowding distance, which prioritizes individuals located in sparsely populated regions of the objective space to preserve solution diversity. The best *N* individuals where *N* is the original population size were selected from the top-ranked fronts to form the population for the next generation. This elitist selection strategy ensures that the most competitive solutions are retained, while also allowing new and diverse candidates to contribute to the ongoing evolutionary process.

In step 6, the optimization process continued iteratively through multiple generations, with each cycle involving evaluation, non-dominated sorting, variation and selection. The process was terminated once a predefined stopping criterion was reached. In this study, the termination condition was set to a fixed number of 50 generations. At the conclusion of the optimization, the final population represented a diverse set of Pareto-optimal solutions, illustrating the trade-off frontier between the two mechanical objectives: tensile strength and Shore D hardness. To further analyze the results, the predicted tensile strength and Shore D hardness values were normalized using min–max scaling and combined to compute a composite performance score, facilitating the identification of well-balanced formulations. The resulting solutions were visualized as a Pareto front, enabling clear decision-making based on performance trade-offs.

In conclusion, the NSGA-II algorithm was effectively implemented to address the multi-objective optimization problem inherent in the design of PLA/SCG/Silane bio-composites. The optimization process was configured with a population size of 50 and run for 50 generations, providing a practical balance between search space exploration and computational efficiency. The genetic operators were set as follows. Uniform crossover was applied with a probability of 0.5. Uniform integer mutation was configured with a global mutation probability of 0.4 and a per-gene mutation probability of 0.2. These operators were selected to maintain sufficient genetic diversity across the population and to explore the search space thoroughly. Selection and survival strategies were based on non-dominated sorting and crowding distance, ensuring both convergence toward the Pareto front and the preservation of solution diversity. Importantly, the evaluation of each candidate solution was performed using a pre-trained XGBoost surrogate model, which enabled rapid approximation of mechanical performance without the need for additional physical testing. Together, these parameter settings and algorithmic choices enabled the NSGA-II framework to efficiently identify a wide range of optimal formulations that balance the dual objectives of tensile strength and Shore D hardness.

The resulting Pareto front serves as a valuable design tool for the future development of bio-composites with tailored mechanical properties. Such a framework can accelerate material formulation decisions in applications requiring trade-offs between strength and durability, such as structural packaging or biodegradable consumer products.

### 3.6 Definition and calculation of composite score

To enable quantitative ranking of the Pareto-optimal solutions, a composite score (CS) was introduced as a normalized performance index that integrates both tensile strength (TS) and Shore D hardness (HD) into a single metric representing the overall mechanical performance of each composite formulation.

Each property (tensile strength and Shore D hardness) was normalized using the min–max method as shown in eqn (20):20
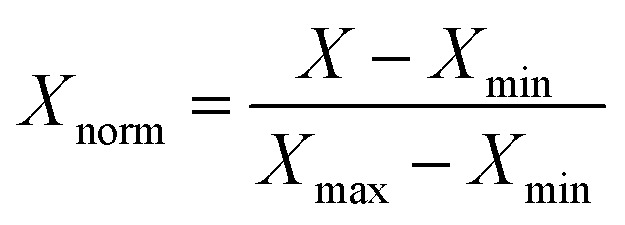
where *X* represents the measured value of tensile strength or hardness, and *X*_min_ and *X*_max_ are the minimum and maximum values of the corresponding property among all experimental and optimized formulations.

The composite score was then calculated as the sum of the two normalized properties according to eqn (21):

21 CS = *X*_TS,norm_ + *X*_HD,norm_

A higher CS value indicates a better combined mechanical performance, reflecting the formulation that simultaneously achieves high tensile strength and high hardness. This normalization procedure ensures that both mechanical properties contribute equally and objectively to the evaluation, avoiding any unit bias or dominance of one property over the other.

## 4 Conclusions

This study demonstrated an integrated data-driven framework for optimizing the mechanical properties of PLA/SCG/Silane bio-composites through the combination of synthetic data generation, XGBoost regression modeling, and NSGA-II multi-objective optimization. Experimental testing confirmed that material composition strongly influenced both tensile strength and Shore D hardness, where excessive SCG content reduced strength, while moderate silane addition enhanced interfacial bonding and overall mechanical performance.

By augmenting the original dataset with 159 synthetic samples, the XGBoost model achieved improved predictive accuracy, with *R*^2^ values of 0.884 and 0.908 and MSEs of 12.64 and 0.071 for tensile strength and hardness, respectively. The enhanced surrogate model, integrated within the NSGA-II algorithm, effectively explored the design space and produced well-distributed Pareto-optimal solutions. The optimal formulation, comprising 1490 g PLA, 121 g SCG, and 20 g silane, yielded a tensile strength of 53.33 MPa and a Shore D hardness of 80.06, representing the best balance between strength and hardness.

Overall, the proposed XGBoost NSGA-II framework offers a scalable and computationally efficient pathway for data-driven bio-composite design, reducing experimental effort and material waste while delivering superior mechanical performance. Future work should focus on extending this framework to incorporate additional bio-fillers, real-scale manufacturing validation, and deep learning-based modeling to capture more complex material interactions and cost-performance trade-offs for industrial application. It should be noted that the proposed optimal formulation was derived from the model's prediction, and its experimental validation remains a subject for future investigation to further confirm the reliability and applicability of the developed optimization framework.

## Author contributions

Atthaphon Ariyarit: validation, supervision, resources, project administration, methodology, investigation, formal analysis, data curation, conceptualization. Attasit Wiangkham: writing – review & editing, writing – original draft, visualization, validation, software, resources, methodology, investigation, formal analysis, data curation, conceptualization. Phatthawit Siripaiboonsub: writing – original draft, resources, methodology, investigation, formal analysis, data curation. Jittiwat Nithikarnjanatharn: resources, methodology, investigation, formal analysis, data curation, Wannisa Nutkhum: resources, methodology, investigation, formal analysis, data curation. Prasert Aengchuan: writing – review & editing, visualization, validation, supervision, resources, project administration, methodology, investigation, formal analysis, data curation, conceptualization.

## Conflicts of interest

The authors declare that they have no known competing financial interests or personal relationships that could have appeared to influence the work reported in this paper.

## Data Availability

Data will be made available on request. Supplementary information: data, tables and figures. See DOI: https://doi.org/10.1039/d5ra06825h.
